# Exploring the relationship between air pollution, non-alcoholic fatty liver disease, and liver function indicators: a two-sample Mendelian randomization analysis study

**DOI:** 10.3389/fendo.2024.1396032

**Published:** 2024-11-29

**Authors:** Qingliang Song, Jinyue Pan, Maoxing Pan, Chuiyang Zheng, Wen Fan, Jianwei Zhen, Dajin Pi, Zheng Liang, Haiyan Shen, Yuanyou Li, Qinhe Yang, Yupei Zhang

**Affiliations:** School of Traditional Chinese Medicine, Jinan University, Guangzhou, Guangdong, China

**Keywords:** air pollution, non-alcoholic fatty liver disease, liver function indicators, PM2.5, causal relationship, Mendelian randomization study

## Abstract

**Background and aims:**

Non-alcoholic fatty liver disease (NAFLD) is a common metabolic disorder worldwide, with an increasing incidence in recent years. While previous studies have suggested an association between the air pollutant PM2.5 and NAFLD, there is still considerable debate regarding the existence of a clear causal relationship between air pollution and NAFLD. This study aims to employ Mendelian randomization methods to evaluate the causal relationship between major air pollutants and NAFLD.

**Method:**

We conducted Mendelian randomization analyses on a large-scale publicly available genome-wide association study (GWAS) dataset of European populations to dissect the association between air pollutants, NAFLD, and liver function indicators. We used five different analysis methods, including Inverse-variance weighted (IVW), Weighted median, MR-Egger, Simple mode, and Weighted mode, to analyze the data. We also tested for pleiotropy, heterogeneity, and sensitivity of the results.

**Results:**

This study utilized four common exposures related to air pollution and four outcomes related to NAFLD. The results regarding the association between air pollutants and NAFLD (PM2.5: *P*=0.808, 95% CI=0.37-3.56; PM10: *P*=0.238, 95% CI=0.33-1.31; nitrogen dioxide: *P*=0.629, 95% CI=0.40-4.61; nitrogen oxides: *P*=0.123, 95% CI=0.13-1.28) indicated no statistically significant correlation between them. However, notably, there was a causal relationship between PM10 and serum albumin (ALB) levels (*P*=0.019, 95% CI=1.02-1.27).

**Conclusion:**

This MR study found no evidence of a causal relationship between air pollution and NAFLD in European populations. However, a statistically significant association was observed between PM10 and ALB levels, suggesting that the air pollutant PM10 may impact the liver’s ability to synthesize proteins.

## 1 Introduction

Non-alcoholic fatty liver disease (NAFLD) is a clinical-pathological syndrome characterized by hepatocellular steatosis and lipid accumulation (1). It includes a range of liver abnormalities, starting from simple steatosis (NAFL) to non-alcoholic steatohepatitis (NASH), with various disease progression patterns that can result in liver fibrosis, cirrhosis, and cancer (2, 3). Epidemiological studies have shown a strong correlation between NAFLD and metabolic diseases such as obesity, diabetes, hypertension, and dyslipidemia (4), leading many scholars in recent years to refer to it as metabolic dysfunction-associated fatty liver disease (MAFLD) to emphasize the impact of metabolism on the disease (5–7).

Currently, experts estimate that NAFLD affects around 25% of the global population, and there has been a rising trend in recent years (8). With its increasing prevalence, NAFLD has become a significant public health concern globally. Despite the high medical demand for NAFLD, no effective drugs targeting NAFLD have yet received approval from the United States Food and Drug Administration (FDA) and the National Medical Products Administration (NMPA) (9), making lifestyle modifications still a recommended intervention (10). Therefore, it is crucial to identify factors that may influence the occurrence and progression of NAFLD and implement effective interventions to reduce the incidence of NAFLD.

Air pollutants primarily originate from human activities or natural events, including pollutants from burning fossil fuels and sources from natural disasters, mainly comprising particulate matter (PM2.5, PM10), sulfur dioxide, nitrogen dioxide, ozone, and nitrogen oxides (11). Prolonged exposure to air pollutants is bound to have adverse effects on human health. Evidence suggests that long-term exposure to air pollution or fine particulate matter PM2.5 can negatively impact human health, increasing the risk of cardiovascular events and diseases such as diabetes (12–15). Furthermore, prolonged environmental exposure to fine particulate matter PM2.5 may be associated with an increased risk of NAFLD development (16–18). However, these reports still face numerous contradictions and controversies (19–21), necessitating further investigation and validation. Based on this, we hypothesize: Is there a causal relationship between air pollutants and NAFLD?

Mendelian randomization (MR) is a widely used analytical method for exploring causal relationships. Based on the random allocation of genes from parents to offspring, MR uses differences in human genotypes as instrumental variables (IVs) to investigate the causal impact of exposures on outcomes (22). MR can minimize confounding factors to a great extent as genetic variations are randomly allocated to offspring and thus independent of environmental factors, which are typically confounders associated with exposure and outcome (23). In conclusion, well-designed MR studies can provide more reliable evidence to guide clinical practice (24, 25).

In this study, we utilized a large amount of publicly available GWAS data and conducted a two-sample MR analysis to elucidate the impact of air pollutants on the development of NAFLD, thereby further investigating the causal relationship between air pollution and NAFLD, providing new insights for NAFLD prevention.

## 2 Methods

### 2.1 Study design

Our design is based on the three core assumptions of MR (26): assumption 1, the relevance assumption: strong associations exist between genetic variations and exposure factors; assumption 2, the independence assumption: genetic variations are independent of confounding factors that influence both exposure and outcome; assumption 3, the exclusion restriction assumption: genetic variations only affect outcomes through exposure and not through other pathways.

**Figure 1:**
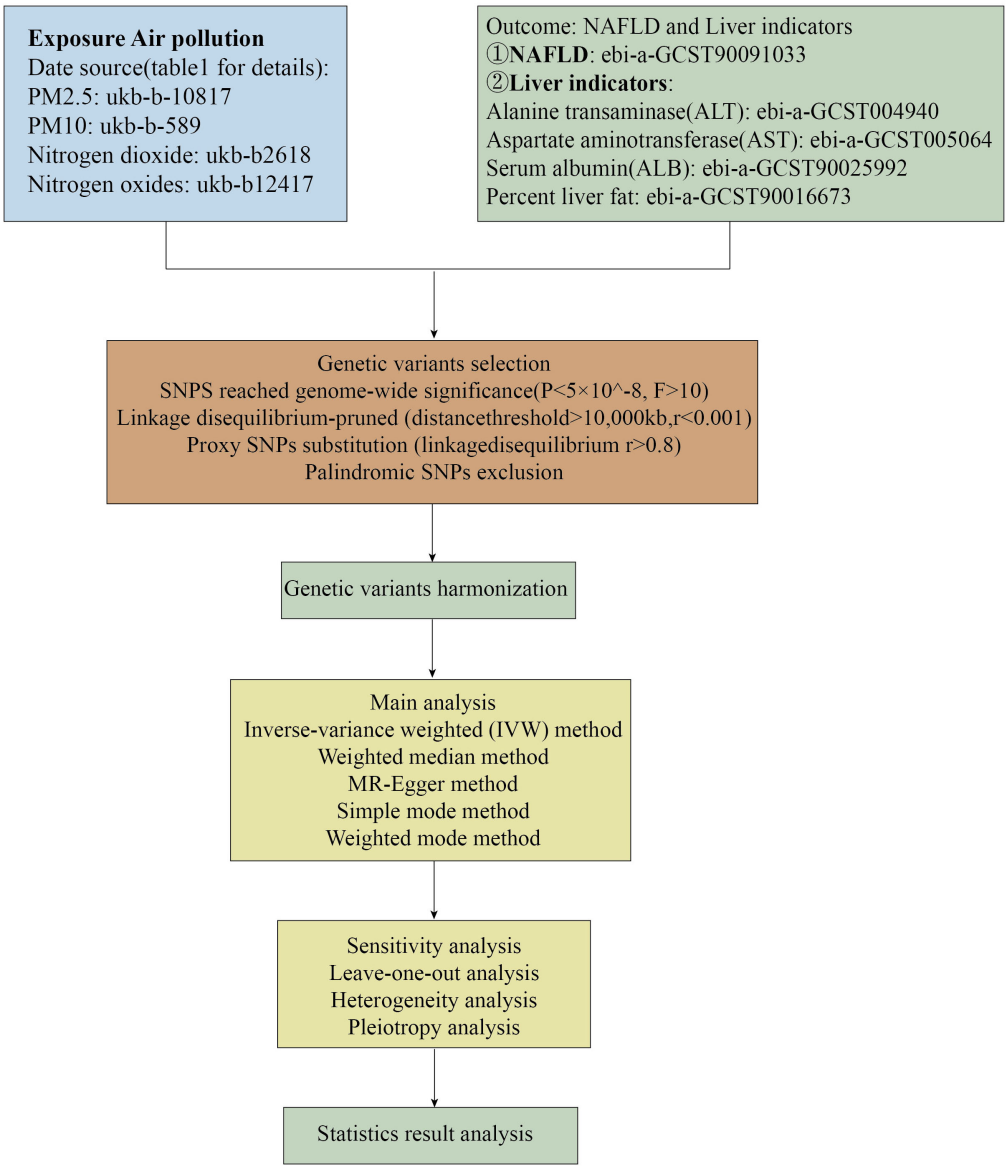
Flow chart of this Mendelian randomization study.

**Figure 2:**
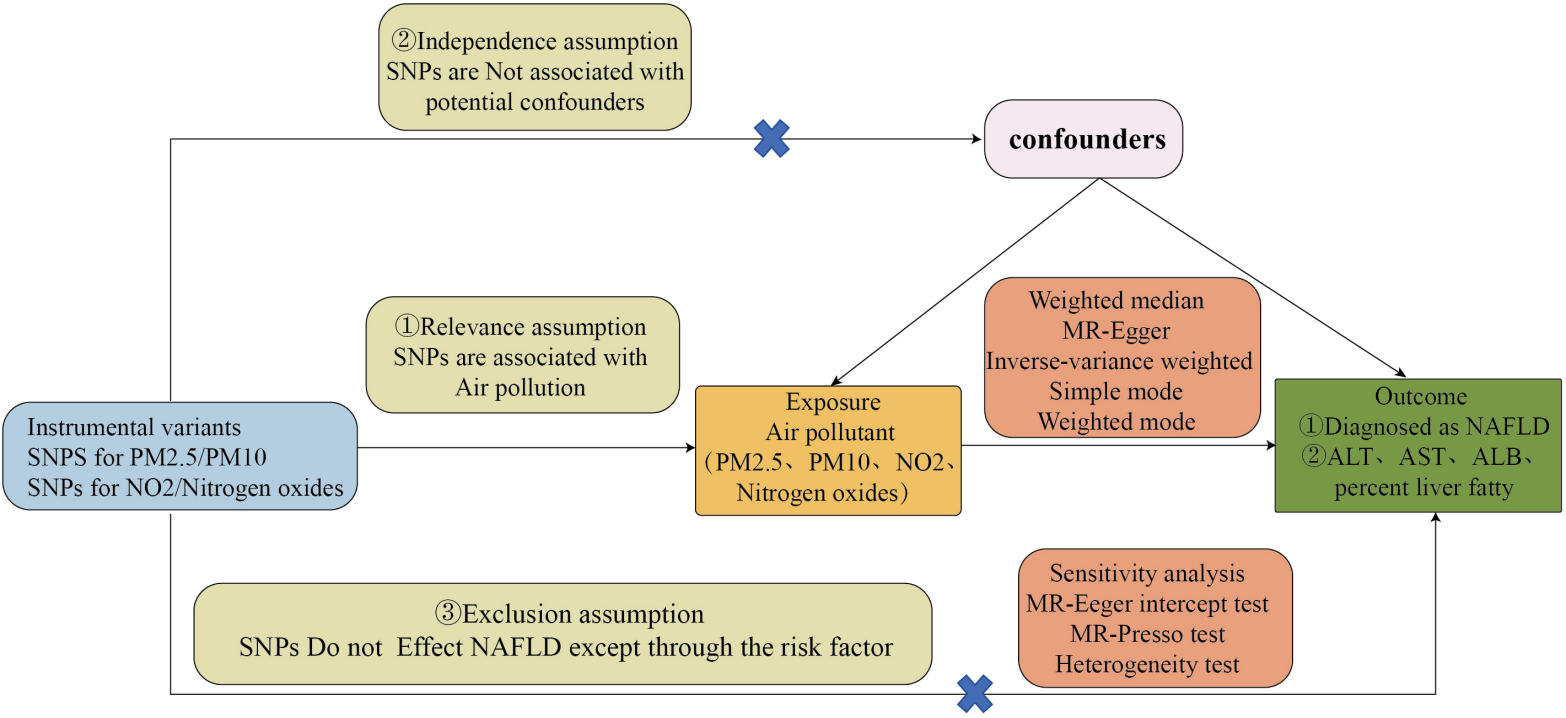
Basic assumptions of Mendelian randomization and main design of this study.

We utilized common air pollution indicators, namely PM2.5, PM10, nitrogen dioxide, and nitrogen oxides, as exposure factors. A diagnosis of Non-Alcoholic Fatty Liver Disease (NAFLD) was considered the outcome of Mendelian randomization analysis. Furthermore, given the primary characteristics of NAFLD are hepatic steatosis and liver dysfunction, we conducted a second Mendelian randomization analysis on alanine aminotransferase (ALT) levels, aspartate aminotransferase (AST) levels, serum albumin (ALB) levels, and liver fat percentage in relation to air pollutants to bolster the persuasiveness of our findings. The causal relationship between air pollution and NAFLD was assessed through two-sample Mendelian randomization analyses. The flowchart of the Mendelian randomization study and the fundamental hypotheses of this research are depicted in **Figures 1**, **2**, respectively.

### 2.2 Data sources

The data used in this study were obtained from the Open GWAS database, as detailed in **Table 1**. The exposure factors of air pollutants (PM2.5, PM10, nitrogen dioxide, nitrogen oxides) were sourced from a prospective study involving over half a million participants in the UK, with phenotype and genetic details already published. We utilized European population GWAS samples for the study, including PM2.5 (GWAS ID: ukb-b-10,817), PM10 (GWAS ID: ukb-b-589), nitrogen dioxide (GWAS ID: ukb-b-2,618), and nitrogen oxides (GWAS ID: ukb-b-12,417). Additionally, NAFLD and liver indicators (ALT, AST, ALB, percent liver fat) were used as outcome measures, sourced from European populations: NAFLD (GWAS ID: ebi-a-GCST90091033), ALT (GWAS ID: ebi-a-GCST004940), AST (GWAS ID: ebi-a-GCST005064), ALB (GWAS ID: ebi-a-GCST90025992), Percent liver fat (GWAS ID: ebi-a-GCST90016673). Specific SNP information and corresponding R^2^ and F-statistics, are shown in **Supplementary Tables 1**–**5**.

**Table 1:** Summary of the genome-wide association studies (GWAS) included in this two-sample MR study.

Exposures/outcomes	Dataset	Sample size	Number of SNPs	Population	Consortium	Sex	Years
Particulate matter (PM2.5)	ukb-b-10,817	423,796	9,851,867	European	MRC-IEU	Males and Females	2018
Particulate matter (PM10)	ukb-b-589	455,314	9,851,867	European	MRC-IEU	Males and Females	2018
Nitrogen dioxide (NO2)	ukb-b-2,618	456,380	9,851,867	European	MRC-IEU	Males and Females	2018
Nitrogen oxides	ukb-b-12,417	456,380	9,851,867	European	MRC-IEU	Males and Females	2018
NAFLD	ebi-a-GCST90091033	778,614	6,784,388	European	NA	Males and Females	2021
ALT	ebi-a-GCST004940	9,731	16,987,168	European	NA	Males and Females	2017
AST	ebi-a-GCST005064	9,463	18,153,643	European	NA	Males and Females	2017
Percent liver fat	ebi-a-GCST90016673	32,858	9,275,407	European	NA	Males and Females	2021
ALB	ebi-a-GCST90025992	400938	4219040	European	NA	Males and Females	2021

"NA" usually refers to "Not Available" or "Not Applicable". This indicates that a data point has no available data or is not applicable in a specific situation.

### 2.3 Selection of instrumental variables

We employed the following steps to select valid SNPs (1): setting the genome-wide significance level at *P* < 5×10^−8^ to meet the first key assumption that these SNPs are significantly associated with the exposure (2). Linkage disequilibrium clustering (r^2^ < 0.001, region size = 10,000 kb) to ensure the independence of SNPs (3). Interpretation and strength of R^2^ and F-statistic tests to eliminate low-strength SNPs (F-statistic < 10). R^2^ = 2×EA×(1−EAF)×betaˆ2/(2×EAF×(1- EAF) × betaˆ2) + 2×EAF×(1−EAF) × SE×N×betaˆ2, F = R^2^×(N−2)/(1−R^2^) (4). Utilizing PhenoScanner V2 to query SNP phenotypes when necessary, excluding SNPs closely related to confounding factors to meet the second assumption of exclusivity (27). Initially setting the significance level at *P* < 5×10^−6^ revealed the presence of outliers and horizontal pleiotropy, with further analysis indicating no causal relationship between the two. To enhance result accuracy, we decided to uniformly set the *P*-value at *P* < 5×10^−8^, significantly reducing outliers and addressing pleiotropy, ensuring result reliability without altering statistical outcomes.

### 2.4 Mendelian randomization analysis

In this study, we employed five methods for data analysis, including Weighted median, MR-Egger, IVW, Simple mode, and Weighted mode. Among these, the IVW method played a predominant role. IVW is the primary method for conducting MR analysis, as it is the most commonly used and convincing MR statistical method when SNPs are valid and show no evidence of pleiotropy (28). The IVW test selects a fixed or random effects model based on the presence of heterogeneity. The MR-Egger method allows for the intercept of the regression line to vary in the presence of pleiotropy in the IVs. It assesses the magnitude of pleiotropy between IVs using the intercept, while the slope serves as an estimate of the causal effect, providing consistent estimates even when all instrumental variables exhibit genetic pleiotropy (29). The strength of the Weighted median method lies in its ability to consistently estimate causal relationships even with over 50% of invalid instrumental variables. Therefore, the study utilized MR-Egger regression and Weighted median as complementary methods. A significance level of *P* < 0.05 was considered statistically significant.

### 2.5 Sensitivity analysis

Furthermore, we conducted analyses on pleiotropy, heterogeneity, and sensitivity. Heterogeneity testing using Cochran’s Q statistic, with no significant heterogeneity among the instrumental variables, led IVW to adopt fixed effects models uniformly. Outlier detection using the MR-PRESSO method revealed *P* > 0.05, indicating no outliers were detected. Horizontal pleiotropy testing with MR-Egger showed no evidence of horizontal pleiotropy, with *P* > 0.05 (**Table 2**). Additionally, stability assessment of MR results through leave-one-out analysis indicated that no single SNP significantly influenced the stability of the study results. Therefore, the MR results on the association between air pollutants and NAFLD and its liver indicators were deemed reliable.

**Table 2:** Mendelian randomization (MR) analysis of air pollution (particulate matter, nitrogen dioxide, and nitrogen oxides, exposure) with Liver Indicators in NAFLD in the European population (IVW method).

Exposures	Outcomes (biomarkers)	Beta (95% CI)	*P*	Number of SNPs	*R* ^2^	*F*	*P (Cochran’s Q heterogeneity test)*	*P (MR-PRESSO global test)*	*P (MR-Egger intercept test)*
Particulate matter (PM2.5)	Alanine transaminase (ALT)	0.319(0.75,2.57)	0.317	2	0.014%	30.058	NA	NA	NA
	Aspartate aminotransferase (AST)	0.808(0.75,6.76)	0.150	1	0.008%	33.149	0.060	NA	NA
	Serum albumin (ALB)	-0.473(0.38,1.04)	0.068	2	0.024%	49.979	0.011	NA	NA
	Percent liver fat	0.217(0.80,1.92)	0.331	7	0.061%	36.783	0.032	0.644	0.781
Particulate matter (PM10)	Alanine transaminase (ALT)	0.033(0.70,1.52)	0.869	6	0.689%	44.727	0.021	0.070	0.289
	Aspartate aminotransferase (AST)	-0.221(0.60,1.08)	0.143	10	0.103%	38.972	0.050	0.059	0.987
	Serum albumin (ALB)	0.131(1.02,1.27)	0.019	9	0.087%	43.911	0.450	0.522	0.574
	Percent liver fat	-0.270(0.58,1.00)	0.051	22	0.178%	36.834	0.431	0.467	0.478
Nitrogen dioxide (NO2)	Alanine transaminase (ALT)	0.163(0.37,3.76)	0.784	2	0.015%	34.024	0.042	NA	NA
	Aspartate aminotransferase (AST)	0.402(0.68,3.28)	0.317	1	0.007%	33.273	NA	NA	NA
	Serum albumin (ALB)	-0.334(0.38,1.34)	0.298	3	0.034%	38.846	7.823	NA	0.740
	Percent liver fat	-0.079(0.45,1.91)	0.830	5	0.042%	38.370	0.122	0.171	0.116
Nitrogen oxides	Alanine transaminase (ALT)	0.442(0.95,2.56)	0.081	2	0.014%	32.108	0.622	NA	NA
	Aspartate aminotransferase (AST)	0.687(0.97,4.07)	0.060	1	0.007%	33.405	NA	NA	NA
	Serum albumin (ALB)	-0.357(0.42,1.13)	0.143	3	0.028%	42.126	0.001	NA	0.921
	Percent liver fat	-0.034(0.64,1.48)	0.868	8	0.062%	35.466	0.539	0.555	0.940

"NA" usually refers to "Not Available" or "Not Applicable". This indicates that a data point has no available data or is not applicable in a specific situation.

### 2.6 Statistical analysis

All analyses were conducted using the “TwoSampleMR” and “MR-PRESSO” packages in R version 4.2.2. The statistical significance threshold for evidence was set at *P* < 0.05.

## 3 Results

### 3.1 Air pollutants and NAFLD

**Figure 3:**
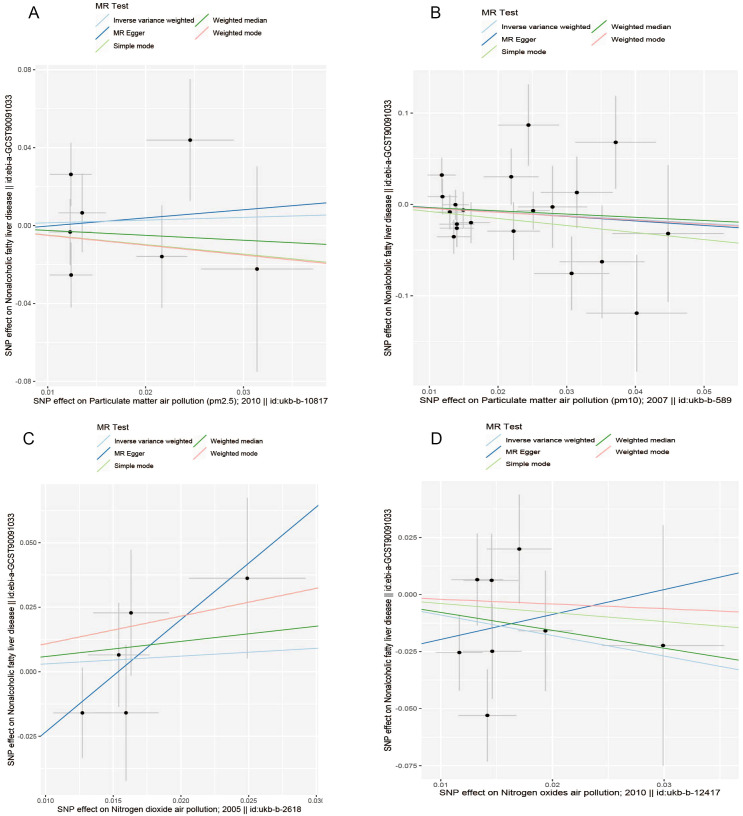
Scatter plots for causal single nucleotide polymorphism (SNP) effect of air pollution (particulate matter, nitrogen dioxide, and nitrogen oxides) on NAFLD in the European population. We plot each black point to represent each SNP on the exposure (horizontal axis) and the outcome (vertical axis), with error bars corresponding to each standard error (SE). The slope of each line corresponds to the combined estimate using each method of the inverse variance weighted (light blue line), the MR-Egger (blue line), the simple mode (light green line), the weighted median (green line), and the weighted mode (pink line). **(A)** PM2.5; **(B)** PM10; **(C)** Nitrogen dioxide; **(D)** Nitrogen oxides.

**Figure 4:**
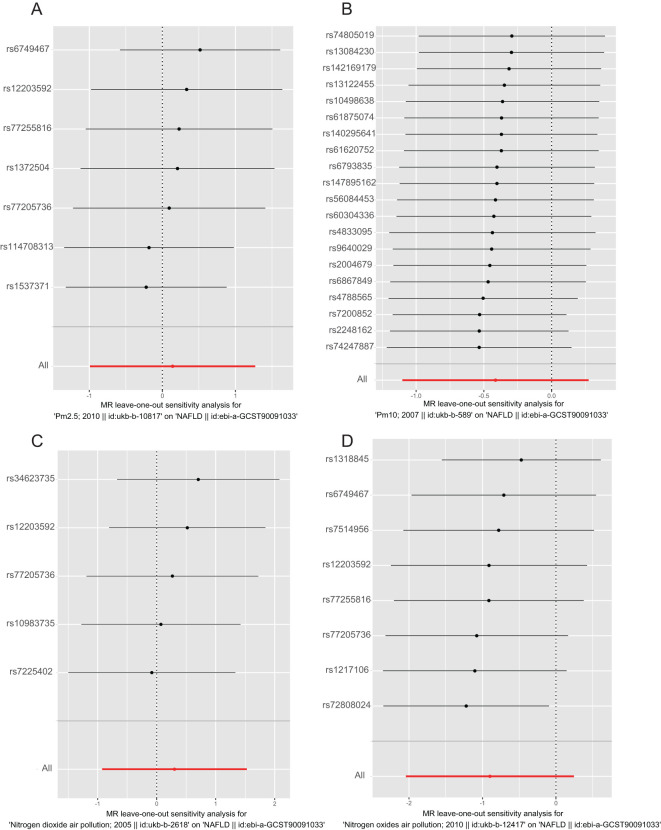
Leave-one-out analysis plot for causal SNP effect of air pollution (particulate matter, nitrogen dioxide, and nitrogen oxides) on NAFLD in the European population. The error bars indicate the 95% confidence interval (CI). **(A)** PM2.5; **(B)** PM10; **(C)** Nitrogen dioxide; **(D)** Nitrogen oxides.

**Figure 5:**
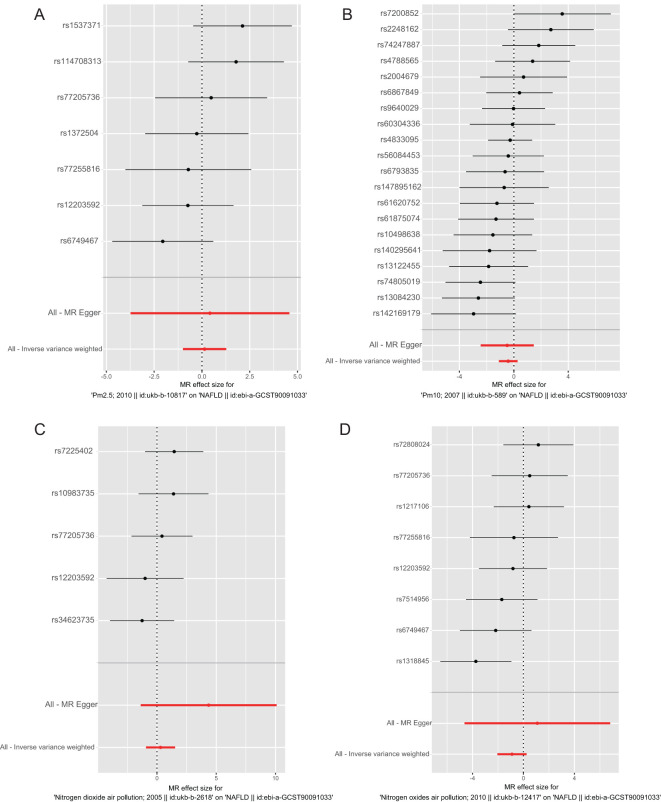
Forest plots for causal SNP effect of air pollution (particulate matter, nitrogen dioxide, and nitrogen oxides) on NAFLD in the European population. The error bars indicate the 95% confidence interval (CI). **(A)** PM2.5; **(B)** PM10; **(C)** Nitrogen dioxide; **(D)** Nitrogen oxides.

**Figure 6:**
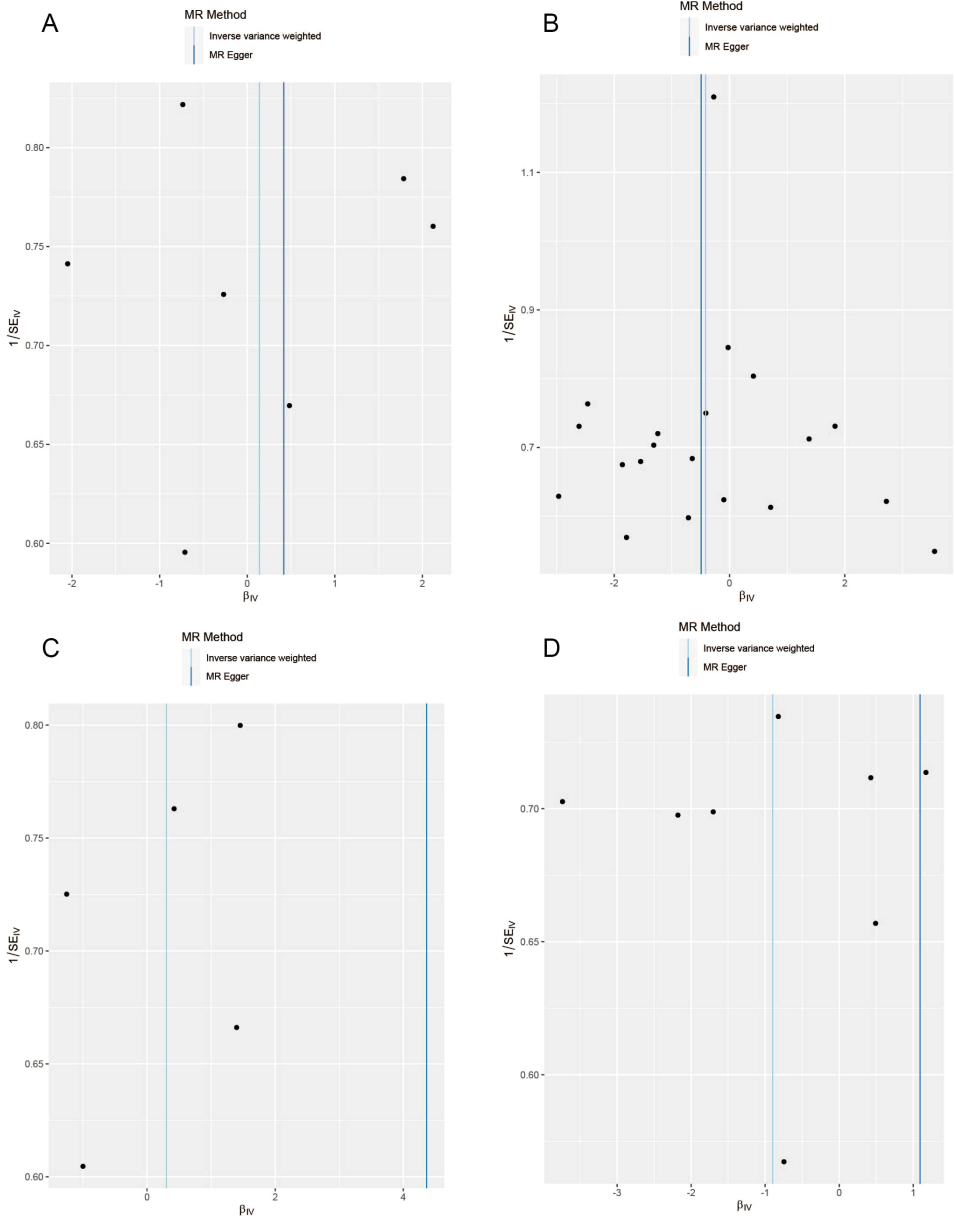
Funnel plots for causal SNP effect of air pollution (particulate matter, nitrogen dioxide, and nitrogen oxides) on NAFLD in the European population. **(A)** PM2.5; **(B)** PM10; **(C)** Nitrogen dioxide; **(D)** Nitrogen oxides.

The results of the MR analysis are presented in **Table 3**, along with scatter plots (**Figure 3**), leave-one-out analysis plots (**Figure 4**), forest plots (**Figure 5**), and funnel plots (**Figure 6**). In this study, four common air pollution-related exposures (PM2.5, PM10, nitrogen dioxide, and nitrogen oxides) were used for MR analysis with NAFLD as the outcome. The results between air pollutants and NAFLD showed no statistically significant correlation: PM2.5: *P*=0.808, 95%CI=0.37-3.56; PM10: *P*=0.238, 95%CI=0.33-1.31; nitrogen dioxide: *P*=0.629, 95%CI=0.40-4.61; nitrogen oxides: *P*=0.123, 95%CI=0.13-1.28. The leave-one-out analysis also did not reveal any abnormal SNPs. The corresponding values of R^2^ and F statistics can be found in **Supplementary Table 1**.

**Table 3:** Mendelian randomization (MR) analysis of air pollution (particulate matter, nitrogen dioxide, and nitrogen oxides, exposure) with NAFLD outcome in the European population.

Table Exposures	Outcomes	Methods	Beta	P	Number of SNPs	R^2^	F	*P*(Cochran’s Q heterogeneity test)	*P*(MR-PRESSO global test)	*P*(MR-Egger intercept test)
Particulate matter (PM2.5)	NAFLD	MR-Egger	0.418	0.851	7	0.068%	36.78344	0.278	0.275	0.896
		Weighted median	-0.252	0.727						
		IVW	0.140	0.808						
		Simple mode	-0.488	0.700						
		Weighted mode	-0.504	0.681						
Particulate matter (PM10)		MR-Egger	-0.490	0.628	20	0.164%	37.30839	0.178	0.180	0.935
		Weighted median	-0.352	0.459						
		IVW	-0.414	0.238						
		Simple mode	-0.770	0.353						
		Weighted mode	-0.433	0.540						
Nitrogen dioxide (NO2)		MR-Egger	4.363	0,232	5	0.042%	38.37019	0.511	0.536	0.250
		Weighted median	0.588	0.473						
		IVW	0.302	0.629						
		Simple mode	1.077	0.423						
		Weighted mode	1.077	0.390						
Nitrogen oxides		MR-Egger	1.092	0.721	8	0.062%	35.46614	0.252	0.254	0.512
		Weighted median	-0.784	0.265						
		IVW	-0.899	0.123						
		Simple mode	-0.395	0.764						
		Weighted mode	-0.207	0.860						

### 3.2 Air pollutants and liver indicators

The MR analysis results are presented in **Table 2**. To further investigate the causal relationship between air pollution and NAFLD, we selected several liver indicators closely related to NAFLD (ALT, AST, ALB, percent liver fat). The IVW method results indicated no causal relationship between air pollutants and ALT (PM2.5: *P*=0.317; PM10: *P*=0.869; nitrogen dioxide: *P*=0.784; nitrogen oxides: *P*=0.081), nor between air pollutants and AST (PM2.5: *P*=0.150; PM10: *P*=0.143; nitrogen dioxide: *P*=0.317; nitrogen oxides: *P*=0.060), nor between air pollutants and ALB (PM2.5: *P*=0.068; nitrogen dioxide: *P*=0.298; nitrogen oxides: *P*=0.143), nor between air pollutants and percent liver fat (PM2.5: *P*=0.331; PM10: *P*=0.051; nitrogen dioxide: *P*=0.830; nitrogen oxides: *P*=0.868).

It is worth mentioning that, after controlling for heterogeneity and multiple effects, our study found a statistically significant association between PM10 and ALB (*P*=0.019, 95% CI=1.02-1.27). The corresponding R^2^ and F statistics values can be found in **Supplementary Tables 3–5**.

## 4 Discussion

Previous studies have extensively investigated the causal relationship between air pollution and various diseases and related indicators using Mendelian randomization (MR) methods, such as cardiovascular diseases, diabetes, and thyroid diseases. However, existing evidence regarding the association between air pollution and NAFLD is primarily limited to cross-sectional and cohort studies, which have been subject to considerable controversy and skepticism. Therefore, it is essential to explore the specific relationship between air pollutants and NAFLD, as this understanding would play a crucial role in the early diagnosis and treatment of NAFLD. In this study, we utilized genetic data retrieved from genome-wide association studies (GWAS) databases and systematically evaluated the causal associations between major air pollutants and the onset of NAFLD. Our results revealed no causal relationship between major air pollutants (PM2.5, PM10, nitrogen dioxide, nitrogen oxides) and NAFLD.

Despite the increasing number of studies on the pathogenesis of NAFLD in recent years, the mechanisms underlying its development remain incompletely understood due to its complexity (30). The current pathogenesis of NAFLD is primarily explained by the “double-hit” and “multiple-hit” theories, suggesting that factors such as abnormal lipid metabolism, oxidative stress, inflammatory stimuli, insulin resistance, mitochondrial dysfunction, and disrupted gut microbiota contribute to the occurrence of NAFLD.

Previous research has shown that air pollutants can increase fat inflammation and insulin resistance in diet-induced obese mouse models (31), induce NASH-like phenotypes in mice, impair hepatic glucose metabolism in animal models (32, 33), and disrupt liver glucose and lipid synthesis pathways (34, 35). Clinical studies have demonstrated a significant correlation between air pollution and increased diabetes prevalence in populations, particularly in young, overweight, or obese individuals (36). Air pollution has been identified as a risk factor for type 2 diabetes and is known to promote the development of diabetes and cardiovascular diseases, with cardiovascular diseases being the leading cause of death in NAFLD patients.

Therefore, it is crucial to pay close attention to the impact of air pollution on NAFLD. In contrast to our study, a cross-sectional study (37) analyzed data from 269,705 hospitalized patients diagnosed with NAFLD and estimated average annual PM2.5 exposure using a spatial exposure model to investigate the relationship between environmental PM2.5 exposure and hospitalized patients with NAFLD. The results indicated a significant association between NAFLD and PM2.5 exposure (P< 0.01, 95% CI 1.15-1.33), which was more pronounced in certain populations and regions. Additionally, a cohort study (38) analyzing medical examination data from 2005 to 2017 involving 17,106 hospital patients found a link between long-term environmental PM2.5 exposure and an increased risk of NAFLD, with females, lean individuals, and younger people being more susceptible to the effects of PM2.5. However, these retrospective studies are limited by recall bias and the inclusion of a limited number of cases and geographical regions, rendering their results less reliable.

Therefore, we chose the MR method to conduct gene-level causal analysis of common major air pollutants and NAFLD using single nucleotide polymorphisms (SNPs) closely associated with air pollution as instrumental variables (IVs) to enhance the accuracy and reliability of our study. Our research indicates a lack of statistically significant causal relationships between the current major air pollutants and NAFLD, reducing the likelihood of their clinical relevance and refuting the role of air pollution in the onset of NAFLD. However, in further MR analysis of air pollutants and liver indicators related to NAFLD, we found a statistical association between PM10 and ALB (Beta: 0.131, 95% CI: 1.02-1.27; *P* = 0.019).

Given the current lack of specific biomarkers for NAFLD, its clinical diagnosis primarily relies on liver tissue biopsy (39, 40). However, due to the invasive nature of tissue biopsy, patients’ willingness to undergo liver tissue biopsy is often low, posing challenges to the diagnosis and treatment of NAFLD. Furthermore, due to the heterogeneity of NAFLD, not all NAFLD patients exhibit obesity and high lipids; there are also many lean NAFLD patients. From the perspective of liver enzyme levels, ALT and AST are primarily present within liver cells, and their release into the blood occurs when liver cells are damaged. In patients with mild to moderate NAFLD, due to the strong compensatory capacity of liver cells, their liver enzyme levels may remain within normal ranges, with significant liver enzyme level abnormalities typically seen in more severe cases of NASH. Therefore, it is imperative to approach these results with a rational mindset.

ALB, a crucial protein synthesized by the liver, constitutes approximately 60% of serum proteins and plays a vital role in maintaining acid-base balance, vascular permeability, colloid osmotic pressure, and combating oxidative stress (41–43). Clinically, low albumin levels are often associated with chronic liver disease, malnutrition, and tumors, among others (44). Hypoalbuminemia (<3.5g/dL) is typically the result of hepatocyte death and impaired albumin synthesis due to chronic liver disease. Under inflammatory conditions, ALB levels may also decrease (45, 46). Our study identified a significant association between environmental particulate matter PM10 and ALB (Beta: 0.131, 95% CI: 1.02-1.27; *P*=0.019). Currently, there is a lack of research on the relationship between fine particulate matter PM10 and ALB, but existing studies suggest that environmental PM10 can increase the production of pro-inflammatory cytokines, such as IL-1β and IL-6 (47). To some extent, this may explain our findings that environmental PM10 might affect the synthesis of ALB in liver cells, but further *in vivo* and *in vitro* experiments are needed to validate this hypothesis.

### 4.1 Advantages and limitations

To the best of our knowledge, this is the first Mendelian randomization (MR) study analyzing the relationship between air pollutants and non-alcoholic fatty liver disease (NAFLD) to elucidate the impact of air pollution on NAFLD. The main strength of this study lies in the utilization of large-scale genome-wide association study (GWAS) data for MR analysis, which has increased the sample size and facilitated the identification of reliable causal relationships. The MR-Egger method reduces bias caused by reverse causation and confounding factors. The combination of the inverse-variance weighted (IVW) and MR-Egger methods enhances the reliability of this study.

However, this study also has some limitations. We only used data from individuals of European descent, and further validation is needed to determine if this relationship also exists in other populations. Additionally, although our MR study was based on results with a P-value of 5×10^-8^, only a small number of instrumental variables (IVs) were identified, potentially reducing statistical power. Therefore, despite the F-statistic indicating no clear IV bias, caution should be exercised in interpreting these results. We also tested a threshold of adjusted P-value of 5×10^-6^ for SNP selection, but the results remained unchanged. Lastly, despite the removal of SNPs with confounding factors and the establishment of strict thresholds for horizontal pleiotropy, NAFLD is a broad disease category with challenging clinical diagnosis, and the biological functions of many current genetic variants remain unclear. In addition to natural environmental factors such as air pollutants, social environmental factors, including a patient’s socioeconomic status, income level, and education level, exert varying degrees of influence on NAFLD. Research indicates that a lower socioeconomic status is independently associated with an increased risk of NAFLD (48, 49). Thus, complete avoidance of horizontal pleiotropy may not be achievable.

## 5 Conclusion

In conclusion, our study indicates that major air pollutants (PM2.5, PM10, nitrogen dioxide, and nitrogen oxides) do not show a clear causal relationship with NAFLD. Furthermore, we are excited to report a statistically significant association between environmental particulate matter PM10 and ALB, but further experimental and mechanistic studies are needed to explore this relationship in depth.

## Data Availability

The original contributions presented in the study are included in the article/**Supplementary Material**. Further inquiries can be directed to the corresponding authors.
