# Supplementary material for: Exploring the relationship between air pollution, non-alcoholic fatty liver disease, and liver function indicators: a two-sample Mendelian randomization analysis study
Source: Front Endocrinol (Lausanne). 2024 Nov 29;15:1396032. doi: 10.3389/fendo.2024.1396032 (PMC11637881; doi:10.3389/fendo.2024.1396032)
Supplement: Supplementary file 1 [file Table1.docx]

Supplementary Material

## Supplementary Tables

**Supplementary Table 1.** Genetic variants significantly associated with air pollution exposure as instrumental variables and NAFLD as outcomes.

| SNP | Effect allele | Other allele | Beta | SE | EAF | *P* | F | R^2^ |
| --- | --- | --- | --- | --- | --- | --- | --- | --- |
| **PM2.5** | | | | | | | | |
| rs114708313 | T | A | 0.024558 | 0.004478 | 0.06585 | 4.20E-08 | 30.07610 | 7.10E-05 |
| rs12203592 | T | C | 0.021666 | 0.002591 | 0.21289 | 6.20E-17 | 69.91779 | 1.65E-04 |
| rs1372504 | A | G | 0.012291 | 0.002219 | 0.37431 | 3.10E-08 | 30.67359 | 7.24E-05 |
| rs1537371 | A | C | 0.012370 | 0.002149 | 0.50014 | 8.50E-09 | 33.14861 | 7.82E-05 |
| rs6749467 | A | G | -0.01239 | 0.002183 | 0.46581 | 1.40E-08 | 32.22833 | 7.60E-05 |
| rs77205736 | T | C | 0.013521 | 0.002413 | 0.27391 | 2.10E-08 | 31.39898 | 7.41E-05 |
| rs77255816 | T | C | 0.031393 | 0.005728 | 0.03651 | 4.20E-08 | 30.04071 | 7.09E-05 |
| **PM10** | | | | | | | | |
| rs10498638 | C | T | 0.014013 | 0.002537 | 0.18832 | 3.30E-08 | 30.51273 | 6.70E-05 |
| rs13084230 | T | C | -0.01356 | 0.002459 | 0.20031 | 3.50E-08 | 30.39510 | 6.68E-05 |
| rs13122455 | T | C | -0.01400 | 0.00246 | 0.19999 | 1.30E-08 | 32.2930 | 7.09E-05 |
| rs140295641 | A | T | -0.03513 | 0.006172 | 0.02735 | 1.30E-08 | 32.40589 | 7.12E-05 |
| rs142169179 | A | G | 0.040185 | 0.007340 | 0.02027 | 4.40E-08 | 29.97080 | 6.58E-05 |
| rs147895162 | C | T | -0.04475 | 0.008107 | 0.01513 | 3.40E-08 | 30.48057 | 6.69E-05 |
| rs2004679 | C | T | 0.011914 | 0.002138 | 0.30768 | 2.50E-08 | 31.04910 | 6.82E-05 |
| rs2248162 | C | T | 0.011818 | 0.002047 | 0.63988 | 7.80E-09 | 33.33063 | 7.32E-05 |
| rs4788565 | A | G | -0.02191 | 0.003994 | 0.06677 | 4.10E-08 | 30.11069 | 6.61E-05 |
| rs4833095 | C | T | 0.025110 | 0.002405 | 0.20694 | 1.70E-25 | 108.9641 | 2.39E-04 |
| rs56084453 | G | A | 0.014935 | 0.002411 | 0.20974 | 5.90E-10 | 38.35160 | 8.42E-05 |
| rs60304336 | T | G | 0.027931 | 0.005029 | 0.04090 | 2.80E-08 | 30.84177 | 6.77E-05 |
| rs61620752 | G | T | 0.016068 | 0.002767 | 0.14834 | 6.4E-09 | 33.71953 | 7.41E-05 |
| rs61875074 | C | A | 0.022266 | 0.003841 | 0.07302 | 6.80E-09 | 33.60045 | 7.38E-05 |
| rs6793835 | A | G | -0.01298 | 0.002238 | 0.26379 | 6.60E-09 | 33.66092 | 7.39E-05 |
| rs6867849 | T | A | -0.03147 | 0.005231 | 0.04047 | 1.80E-09 | 36.19833 | 7.95E-05 |
| rs7200852 | A | C | -0.02442 | 0.004450 | 0.05473 | 4.00E-08 | 30.12925 | 6.62E-05 |
| rs74247887 | T | C | 0.037132 | 0.005880 | 0.02872 | 2.70E-10 | 39.86966 | 8.76E-05 |
| rs74805019 | C | G | -0.03071 | 0.005482 | 0.03371 | 2.10E-08 | 31.38592 | 6.89E-05 |
| rs9640029 | T | C | -0.01380 | 0.001973 | 0.47823 | 2.70E-12 | 48.89763 | 1.07E-04 |
| **Nitrogen dioxide（NO2）** | | | | | | | | |
| rs10983735 | A | G | 0.016311 | 0.002802 | 0.15446 | 5.90E-09 | 33.866665 | 7.42E-05 |
| rs12203592 | T | C | 0.015941 | 0.002408 | 0.21943 | 3.60E-11 | 43.826077 | 9.60E-05 |
| rs34623735 | T | C | 0.012720 | 0.002157 | 0.33449 | 3.70E-09 | 34.774649 | 7.62E-05 |
| rs7225402 | C | T | -0.02489 | 0.004315 | 0.05831 | 8.00E-09 | 33.273019 | 7.29E-05 |
| rs77205736 | T | C | 0.015406 | 0.002269 | 0.27486 | 1.10E-11 | 46.110577 | 1.01E-04 |
| **Nitrogen oxides** | | | | | | | | |
| rs1217106 | G | A | 0.014557 | 0.002519 | 0.78242 | 7.50E-09 | 33.404575 | 7.32E-05 |
| rs12203592 | T | C | 0.01937 | 0.002457 | 0.21944 | 3.20E-15 | 62.163999 | 1.36E-04 |
| rs1318845 | C | T | -0.01417 | 0.002596 | 0.20080 | 4.80E-08 | 29.802152 | 6.53E-05 |
| rs6749467 | A | G | -0.01166 | 0.002096 | 0.46467 | 2.60E-08 | 30.957675 | 6.78E-05 |
| rs72808024 | C | A | -0.01702 | 0.002906 | 0.14844 | 4.60E-09 | 34.335565 | 7.52E-05 |
| rs7514956 | C | A | -0.01461 | 0.002652 | 0.18651 | 3.60E-08 | 30.341420 | 6.65E-05 |
| rs77205736 | T | C | 0.013265 | 0.002315 | 0.27486 | 1.00E-08 | 32.844141 | 7.20E-05 |
| rs77255816 | T | C | 0.029905 | 0.005471 | 0.03692 | 4.60E-08 | 29.879616 | 6.55E-05 |

| SNP | Effect allele | Other allele | Beta | SE | EAF | *P* | F | R^2^ |
| --- | --- | --- | --- | --- | --- | --- | --- | --- |
| **Alanine transaminase(ALT)** | | | | | | | | |
| rs114708313 | T | A | 0.024558 | 0.004478 | 0.06585 | 4.20E-08 | 30.076104 | 7.10E-05 |
| rs77255816 | T | C | 0.031393 | 0.005728 | 0.03651 | 4.20E-08 | 30.040717 | 7.09E-05 |
| **Aspartate aminotransferase(AST)** | | | | | | | | |
| rs1537371 | A | C | 0.012371 | 0.002149 | 0.50014 | 8.50E-09 | 33.148613 | 7.82E-05 |
| **Percent liver fat** | | | | | | | | |
| rs114708313 | T | A | 0.024558 | 0.004477 | 0.06585 | 4.20E-08 | 30.076104 | 7.10E-05 |
| rs12203592 | T | C | 0.021666 | 0.002591 | 0.212894 | 6.20E-17 | 69.917797 | 1.65E-04 |
| rs1372504 | A | G | 0.012291 | 0.002219 | 0.374311 | 3.09E-08 | 30.673597 | 7.24E-05 |
| rs1537371 | A | C | 0.012370 | 0.002148 | 0.500143 | 8.50E-09 | 33.148612 | 7.82E-05 |
| rs6749467 | A | G | -0.01239 | 0.002182 | 0.465814 | 1.40E-08 | 32.228337 | 7.60E-05 |
| rs77205736 | T | C | 0.013521 | 0.002413 | 0.273909 | 2.10E-09 | 31.398980 | 7.41E-05 |
| rs77255816 | T | C | 0.031393 | 0.005727 | 0.036507 | 4.20E-08 | 30.040717 | 7.09E-05 |
| **Serum albumin(ALB)** | | | | | | | | |
| rs12203592 | T | C | 0.021666 | 0.002591 | 0.212894 | 6.20E-17 | 69.917797 | 1.65E-04 |
| rs77255816 | T | C | 0.031394 | 0.005728 | 0.036507 | 4.20E-08 | 30.040717 | 7.09E-05 |

**Supplementary Table 2**. Genetic variants are significantly associated with PM2.5 exposure as instrumental variables and liver indicators as outcomes.

**Supplementary Table 3**. Genetic variants significantly associated with PM10 exposure as instrumental variables and biomarkers as outcomes in the European population.

| SNP | Effect allele | Other allele | Beta | SE | EAF | *P* | F | R^2^ |
| --- | --- | --- | --- | --- | --- | --- | --- | --- |
| **Alanine transaminase(ALT)** | | | | | | | | |
| rs10498638 | C | T | 0.014013 | 0.002536 | 0.188322 | 3.30E-08 | 30.512739 | 6.70E-05 |
| rs114789974 | A | C | -0.05521 | 0.009634 | 0.010467 | 1.00E-08 | 32.832174 | 7.21E-05 |
| rs2004679 | C | T | 0.011914 | 0.002138 | 0.307685 | 2.50E-08 | 31.049100 | 6.82E-05 |
| rs4833095 | C | T | 0.025110 | 0.002405 | 0.206945 | 1.70E-25 | 108.96415 | 2.39E-04 |
| rs6793835 | A | G | -0.01298 | 0.002238 | 0.263796 | 6.60E-09 | 33.660928 | 7.39E-05 |
| rs6867849 | T | A | -0.03147 | 0.005231 | 0.040477 | 1.80E-09 | 36.198336 | 7.95E-05 |
| rs74247887 | T | C | 0.037132 | 0.005880 | 0.028721 | 2.70E-10 | 39.86966 | 8.76E-05 |
| **Aspartate aminotransferase(AST)** | | | | | | | | |
| rs114789974 | A | C | -0.05520 | 0.009634 | 0.01046 | 1.00E-08 | 32.832174 | 7.21E-05 |
| rs140295641 | A | T | -0.03513 | 0.006172 | 0.02735 | 1.30E-08 | 32.405899 | 7.12E-05 |
| rs142169179 | A | G | 0.04018 | 0.00734 | 0.02027 | 4.40E-08 | 29.970805 | 6.58E-05 |
| rs147895162 | C | T | -0.04475 | 0.008107 | 0.01513 | 3.40E-08 | 30.480572 | 6.69E-05 |
| rs4788565 | A | G | -0.02191 | 0.003994 | 0.06677 | 4.01E-08 | 30.110699 | 6.61E-05 |
| rs4833095 | C | T | 0.025110 | 0.002405 | 0.20694 | 1.70E-25 | 108.9641 | 2.39E-04 |
| rs56084453 | G | A | 0.014935 | 0.002411 | 0.20974 | 5.90E-10 | 38.351609 | 8.42E-05 |
| rs60304336 | T | G | 0.027931 | 0.005029 | 0.04090 | 2.80E-08 | 30.841778 | 6.77E-05 |
| rs61620752 | G | T | 0.016068 | 0.002767 | 0.14834 | 6.40E-09 | 33.719538 | 7.41E-05 |
| rs6793835 | A | G | -0.01298 | 0.002238 | 0.26379 | 6.60E-09 | 33.660928 | 7.39E-05 |
| **Percent liver fat** | | | | | | | | |
| rs10498638 | C | T | 0.014013 | 0.002536 | 0.18832 | 3.30E-08 | 30.512739 | 6.70E-05 |
| rs114789974 | A | C | -0.05520 | 0.009634 | 0.01046 | 1.00E-08 | 32.832174 | 7.21E-05 |
| rs13084230 | T | C | -0.01356 | 0.002459 | 0.20031 | 3.50E-08 | 30.395102 | 6.68E-05 |
| rs13122455 | T | C | -0.01400 | 0.002464 | 0.19999 | 1.29E-08 | 32.293040 | 7.09E-05 |
| rs140295641 | A | T | -0.03513 | 0.006172 | 0.02735 | 1.29E-08 | 32.405899 | 7.12E-05 |
| rs142169179 | A | G | 0.040185 | 0.007340 | 0.02027 | 4.39E-08 | 29.970805 | 6.58E-05 |
| rs147895162 | C | T | -0.04475 | 0.008107 | 0.01513 | 3.40E-08 | 30.480574 | 6.69E-05 |
| rs182549 | T | C | -0.01242 | 0.002218 | 0.73882 | 2.10E-08 | 31.357216 | 6.89E-05 |
| rs2004679 | C | T | 0.011914 | 0.002138 | 0.30768 | 2.50E-08 | 31.049100 | 6.82E-05 |
| rs2248162 | C | T | 0.011818 | 0.002047 | 0.63988 | 7.79E-09 | 33.330632 | 7.32E-05 |
| rs4788565 | A | G | -0.02191 | 0.003994 | 0.06677 | 4.09E-08 | 30.110699 | 6.61E-05 |
| rs4833095 | C | T | 0.025110 | 0.002405 | 0.20694 | 1.69E-25 | 108.96415 | 2.39E-04 |
| rs56084453 | G | A | 0.014935 | 0.002411 | 0.20974 | 5.89E-10 | 38.351609 | 8.42E-05 |
| rs60304336 | T | G | 0.027931 | 0.005029 | 0.04090 | 2.80E-08 | 30.841778 | 6.77E-05 |
| rs61620752 | G | T | 0.016068 | 0.002767 | 0.14834 | 6.40E-09 | 33.719538 | 7.41E-05 |
| rs61875074 | C | A | 0.022266 | 0.003841 | 0.07302 | 6.80E-09 | 33.600454 | 7.38E-05 |
| rs6793835 | A | G | -0.01298 | 0.002238 | 0.26379 | 6.59E-09 | 33.660928 | 7.39E-05 |
| rs6867849 | T | A | -0.03147 | 0.005231 | 0.04047 | 1.79E-09 | 36.198336 | 7.95E-05 |
| rs7200852 | A | C | -0.02442 | 0.004450 | 0.05473 | 4.00E-08 | 30.129258 | 6.62E-05 |
| rs74247887 | T | C | 0.03713 | 0.005880 | 0.02872 | 2.69E-10 | 39.869669 | 8.76E-05 |
| rs74805019 | C | G | -0.03071 | 0.005482 | 0.03371 | 2.10E-08 | 31.385927 | 6.89E-05 |
| rs9640029 | T | C | -0.01380 | 0.001973 | 0.47823 | 2.70E-12 | 48.897631 | 1.07E-04 |
| **Serum albumin(ALB)** | | | | | | | | |
| rs114789974 | A | C | -0.05521 | 0.009635 | 0.010467 | 1.00E-08 | 32.83217 | 7.21E-05 |
| rs2004679 | C | T | 0.011915 | 0.002138 | 0.307685 | 2.50E-08 | 31.04910 | 6.82E-05 |
| rs4833095 | C | T | 0.025111 | 0.002406 | 0.206945 | 1.70E-25 | 108.964 | 2.39E-04 |
| rs56084453 | G | A | 0.01494 | 0.002412 | 0.209746 | 5.90E-10 | 38.35160 | 8.42E-05 |
| rs61620752 | G | T | 0.016069 | 0.002767 | 0.148343 | 6.40E-09 | 33.71954 | 7.41E-05 |
| rs7200852 | A | C | -0.02443 | 0.004450 | 0.054732 | 4.00E-08 | 30.12926 | 6.62E-05 |
| rs74247887 | T | C | 0.03713 | 0.005881 | 0.028721 | 2.70E-10 | 39.8697 | 8.76E-05 |
| rs74805019 | C | G | -0.03071 | 0.005482 | 0.033712 | 2.10E-08 | 31.38593 | 6.89E-05 |
| rs9640029 | T | C | -0.0138024 | 0.001974 | 0.478233 | 2.70E-12 | 48.89763 | 1.07E-04 |

**Supplementary Table 4**. Genetic variants significantly associated with NO2 exposure as instrumental variables and biomarkers as outcomes in European population.

| SNP | Effect allele | Other allele | Beta | SE | EAF | *P* | F | R^2^ |
| --- | --- | --- | --- | --- | --- | --- | --- | --- |
| **Alanine transaminase(ALT)** | | | | | | | | |
| rs34623735 | T | C | 0.012720 | 0.002157 | 0.33449 | 3.69E-09 | 34.77464 | 7.62E-05 |
| rs7225402 | C | T | -0.02489 | 0.004315 | 0.058308 | 8.00E-09 | 33.27301 | 7.29E-05 |
| **Aspartate aminotransferase(AST)** | | | | | | | | |
| rs7225402 | C | T | -0.02489 | 0.004315 | 0.058308 | 8.00E-09 | 33.27301 | 7.29E-05 |
| **Percent liver fat** | | | | | | | | |
| rs10983735 | A | G | 0.016311 | 0.002802 | 0.154461 | 5.89E-09 | 33.866665 | 7.42E-05 |
| rs12203592 | T | C | 0.015941 | 0.002408 | 0.219436 | 3.59E-11 | 43.826077 | 9.60E-05 |
| rs34623735 | T | C | 0.012720 | 0.002157 | 0.33449 | 3.69E-09 | 34.774648 | 7.62E-05 |
| rs7225402 | C | T | -0.02489 | 0.004315 | 0.058308 | 8.00E-09 | 33.273018 | 7.29E-05 |
| rs77205736 | T | C | 0.015406 | 0.002268 | 0.274855 | 1.10E-11 | 46.110577 | 1.01E-04 |
| **Serum albumin(ALB)** | | | | | | | | |
| rs10983735 | A | G | 0.016311 | 0.002802 | 0.154461 | 5.87E-09 | 33.866665 | 7.42E-05 |
| rs12203592 | T | C | 0.015941 | 0.002408 | 0.219436 | 3.59E-11 | 43.826077 | 9.60E-05 |
| rs7225402 | C | T | -0.02489 | 0.004315 | 0.058308 | 8.00E-09 | 38.846371 | 1.70E-04 |

| SNP | Effect allele | Other allele | Beta | SE | EAF | *P* | F | R^2^ |
| --- | --- | --- | --- | --- | --- | --- | --- | --- |
| **Alanine transaminase(ALT)** | | | | | | | | |
| rs72808024 | C | A | -0.01702 | 0.002906 | 0.148435 | 4.60E-09 | 34.335565 | 7.52E-05 |
| rs77255816 | T | C | 0.029905 | 0.005470 | 0.036924 | 4.60E-08 | 29.879616 | 6.55E-05 |
| **Aspartate aminotransferase(AST)** | | | | | | | | |
| rs1217106 | G | A | 0.014557 | 0.002518 | 0.782419 | 7.49E-09 | 33.40457 | 7.32E-05 |
| **Percent liver fat** | | | | | | | | |
| rs1217106 | G | A | 0.014557 | 0.002518 | 0.78241 | 7.49E-09 | 33.40457 | 7.32E-05 |
| rs12203592 | T | C | 0.01937 | 0.002456 | 0.21943 | 3.19E-15 | 62.16399 | 1.36E-04 |
| rs1318845 | C | T | -0.01417 | 0.002596 | 0.20079 | 4.79E-08 | 29.80215 | 6.53E-05 |
| rs6749467 | A | G | -0.01166 | 0.002095 | 0.46467 | 2.59E-08 | 30.95767 | 6.78E-05 |
| rs72808024 | C | A | -0.01702 | 0.002906 | 0.14843 | 4.60E-09 | 34.33556 | 7.52E-05 |
| rs7514956 | C | A | -0.01461 | 0.002652 | 0.18651 | 3.59E-08 | 30.34142 | 6.65E-05 |
| rs77205736 | T | C | 0.013264 | 0.002314 | 0.27485 | 1.00E-08 | 32.84414 | 7.20E-05 |
| rs77255816 | T | C | 0.029905 | 0.005470 | 0.03692 | 4.60E-08 | 29.87961 | 6.55E-05 |
| **Serum albumin(ALB)** | | | | | | | | |
| rs12203592 | T | C | 0.01937 | 0.002456 | 0.21943 | 3.19E-15 | 62.16399 | 1.36E-04 |
| rs72808024 | C | A | -0.01702 | 0.002906 | 0.14843 | 4.60E-09 | 34.33556 | 7.52E-05 |
| rs77255816 | T | C | 0.02990 | 0.005470 | 0.03692 | 4.60E-08 | 29.87961 | 6.55E-05 |

**Supplementary Table 5**. Genetic variants significantly associated with nitrogen oxides exposure as instrumental variables and biomarkers as outcomes in European population.
